# Supplementary material for: Flow-Induced Yap/Taz Signaling Balances Endothelial and Hematopoietic Stem Cell Fates
Source: bioRxiv. 2026 Jun 23:2026.06.20.733393. Preprint. [Version 1] doi: 10.64898/2026.06.20.733393 (PMC13374368; doi:10.64898/2026.06.20.733393)
Supplement: 1 — Supplemental Figure 1: Piezo1-YAP axis can be pharmacologically stimulated to drive HSPC production in human HE cells and zebrafish A) qPCR for human YAP target genes in human CD34+ cells exposed to DMSO or GsMTx-4 for 24hrs. Expression normalized to GAPDH housekeeping gene (n = 3, one-way ANOVA, *p ≤ 0.05, **p ≤ 0.01, ***p ≤ 0.001). B) Flow cytometry gating strategy for HLF-tdTomato reporter. C) Flow cytometry gating strategy for human CD34 and CD45 expression. D) Qualitative distribution plot for runx1/cmyb expression WT embryos exposed to DMSO or a dose curve of Piezo1 agonist Yoda1 from ~10ss-36hpf (n ≥ 13 embryos/condition). E) Flow cytometry gating strategy for quantifying zebrafish Flk+/cMyb+ HSPCs in Tg(kdrl:mCherrys916; cmyb:EGFPzf169) transgenic embryos. Supplemental Figure 2: Piezo1 relays cue from hemodynamic stretch to YAP/TAZ signaling A) Single slices of confocal z-stack of fluorescent in situ showing colocalization of piezo1 mRNA (orange) in runx1+ hemogenic endothelial cells (magenta) in the dorsal aorta (cyan) at ~32 hpf. Vasculature is labeled by transgenic GFP expression in Tg(kdrl:EGFP)s843 embryos captured with anti-GFP antibody staining. Scale bar: 5um. B) Quantification of red blood cell circulation (proxy for presence of wall shear stress) in the dorsal aorta at 36hpf. No RBCs/WSS occurs in both 4ng tnnt2a MO and 25uM pimozide treated embryos (n = 3, unpaired Student’s t-test ***p ≤ 0.001; Error bars indicate SD). C) Quantification of heartbeat presence (proxy for source of cyclic stretch-generating pulses) at 36hpf. 25uM pimozide embryos maintain irregular heart contractions, while 4ng tnnt2a MO embryos have none, resulting in a ‘stretch only’ force profile (n = 3; unpaired Student’s t-test ***p ≤ 0.001; Error bars indicate SD). D) WISH for runx1/cmyb shows relatively normal levels of hematopoietic cell production in 36hpf embryos exposed to 25uM pimozide (24–36hpf), compared to decreases in 4ng tnnt2a MO (n ≥ 50 embryos/condition). Scale b [file NIHPP2026.06.20.733393V1-supplement-1.pdf]

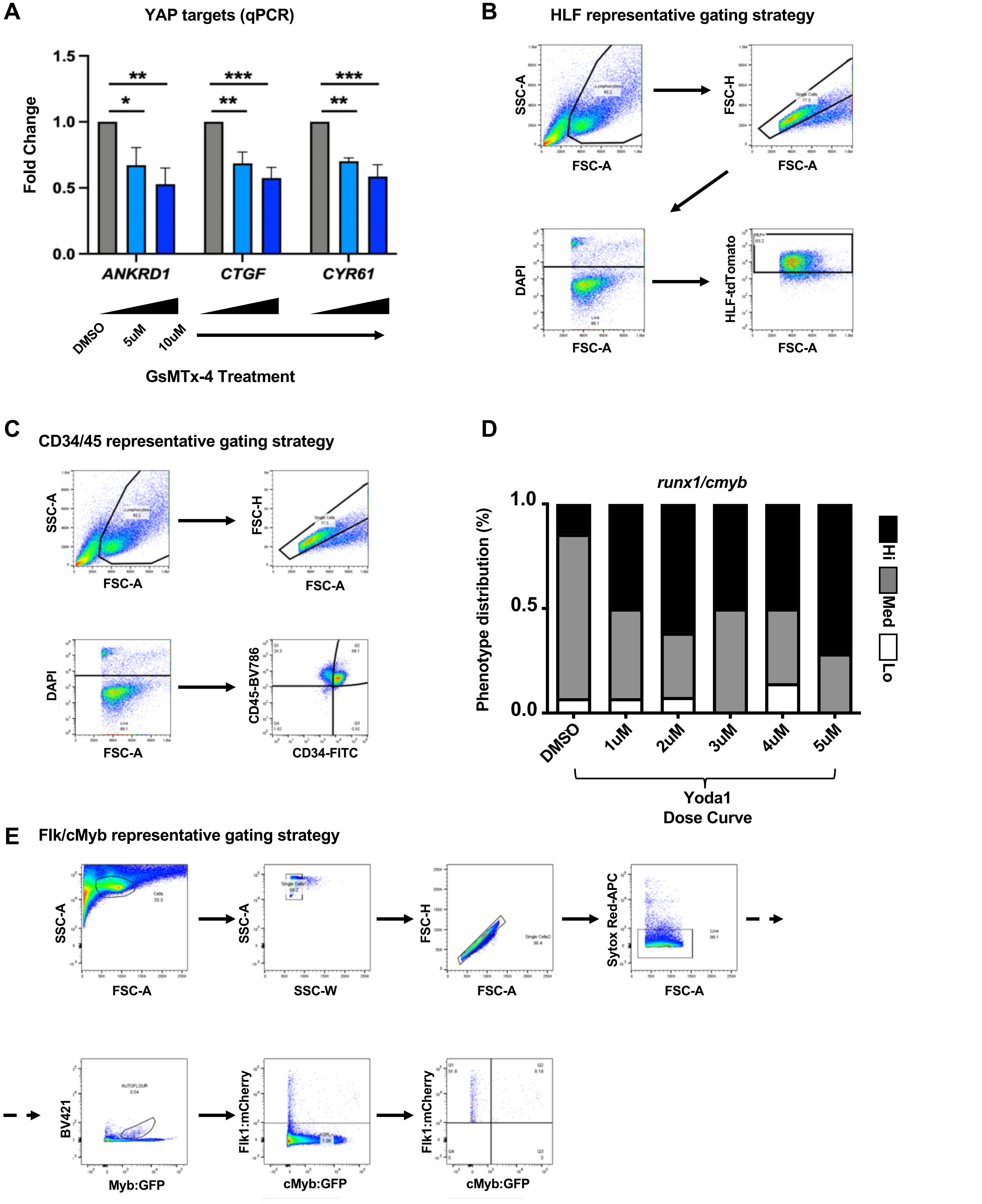
A
YAP targets (qPCR)
Fold Change
1.5
1.0
0.5
0.0
**
*
***
**
***
**
ANKRD1
CTGF
CYR61
DMSO
5uM
10uM
GsMTx-4 Treatment
B
HLF representative gating strategy
SSC-A
FSC-A
FSC-H
FSC-A
DAPI
FSC-A
HLF-tdTomato
FSC-A
C
CD34/45 representative gating strategy
SSC-A
FSC-A
FSC-H
FSC-A
DAPI
FSC-A
CD45-BV786
CD34-FITC
D
runx1/cmyb
Phenotype distribution (%)
1.0
0.5
0.0
Hi
Med
Lo
DMSO
1uM
2uM
3uM
4uM
5uM
Yoda1
Dose Curve
E
Flk/cMyb representative gating strategy
SSC-A
FSC-A
SSC-A
SSC-W
FSC-H
FSC-A
Sytox Red-APC
FSC-A
BV421
Myb:GFP
Flk1:mCherry
cMyb:GFP
Flk1:mCherry
cMyb:GFP

Figure S1

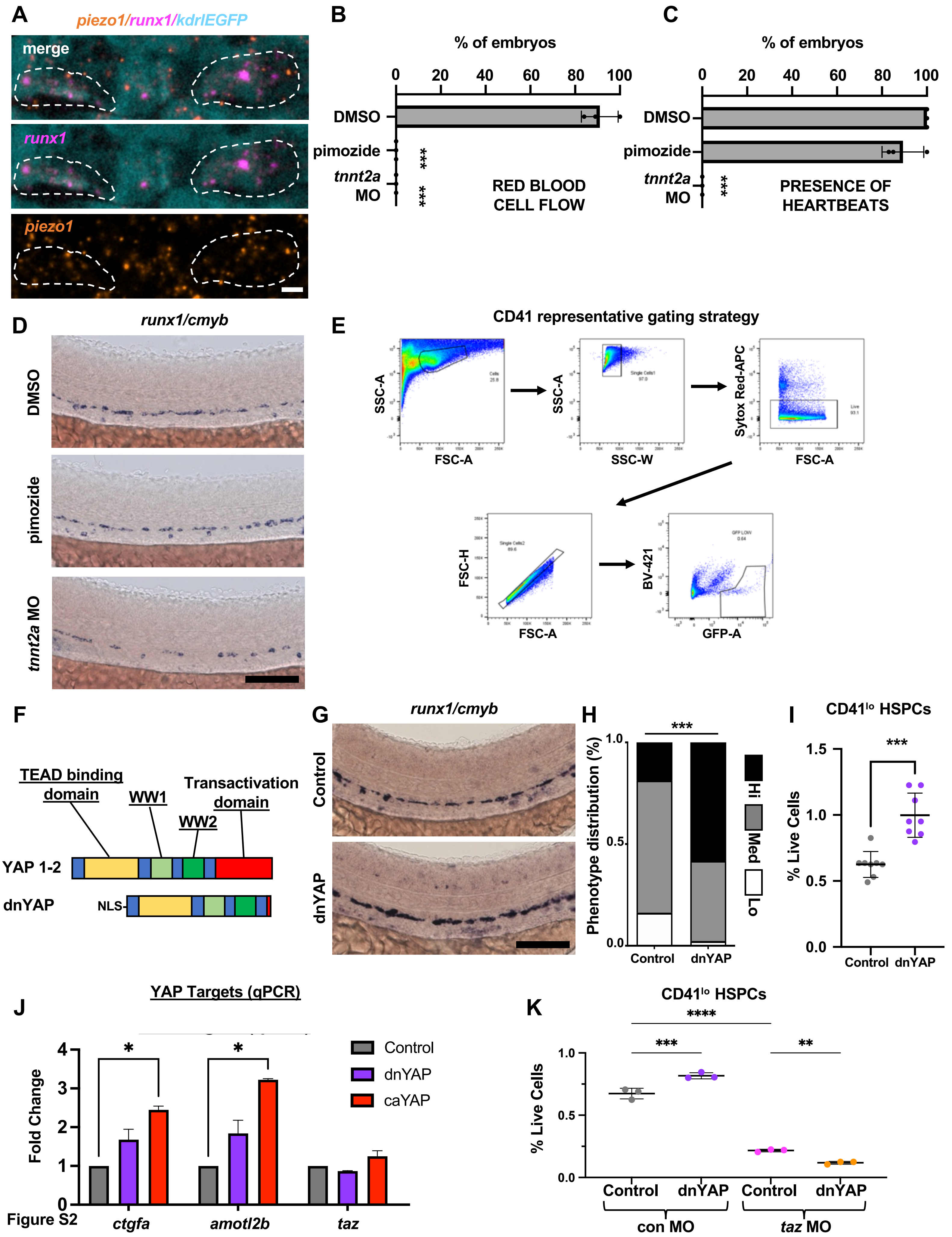

A
piezo1/runx1/kdrlEGFP
merge
runx1
piezo1
B
% of embryos
0 20 40 60 80 100
DMSO
pimozide
tnnt2a MO
***
***
RED BLOOD CELL FLOW
C
% of embryos
0 20 40 60 80 100
DMSO
pimozide
tnnt2a MO
***
PRESENCE OF HEARTBEATS
D
runx1/cmyb
DMSO
pimozide
tnnt2a MO
E
CD41 representative gating strategy
SSC-A
FSC-A
SSC-W
Sytox Red-APC
FSC-H
BV-421
GFP-A
F
TEAD binding domain
WW1
WW2
Transactivation domain
YAP 1-2
dnYAP
NLS
G
runx1/cmyb
Control
dnYAP
H
***
Phenotype distribution (%)
1.0
0.5
0.0
Control
dnYAP
Hi
Med
Lo
I
CD41lo HSPCs
***
% Live Cells
1.5
1.0
0.5
0.0
Control
dnYAP
J
YAP Targets (qPCR)
Fold Change
4
3
2
1
0
*
*
Control
dnYAP
caYAP
ctgfa
amotl2b
taz
K
CD41lo HSPCs
****
***
**
% Live Cells
1.0
0.5
0.0
Control
dnYAP
Control
dnYAP
con MO
taz MO

Figure S2

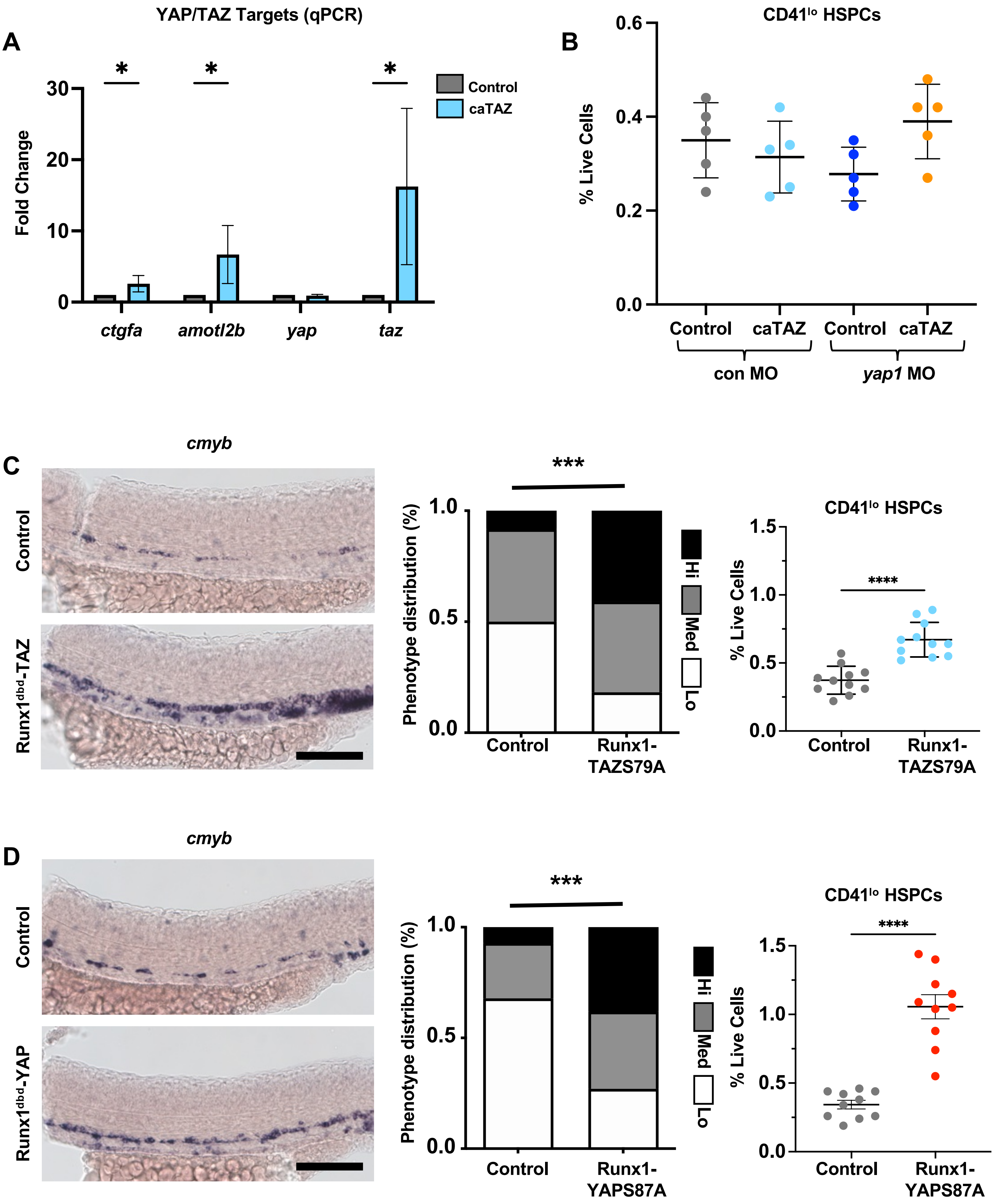

YAP/TAZ Targets (qPCR)
A
Fold Change
Control
caTAZ
ctgfa
amotl2b
yap
taz
CD41lo HSPCs
B
% Live Cells
Control
caTAZ
Control
caTAZ
con MO
yap1 MO
cmyb
C
Control
Runx1dbd-TAZ
Phenotype distribution (%)
Hi
Med
Lo
Control
Runx1-TAZS79A
CD41lo HSPCs
% Live Cells
Control
Runx1-TAZS79A
cmyb
D
Control
Runx1dbd-YAP
Phenotype distribution (%)
Hi
Med
Lo
Control
Runx1-YAPS87A
CD41lo HSPCs
% Live Cells
Control
Runx1-YAPS87A

Figure S3

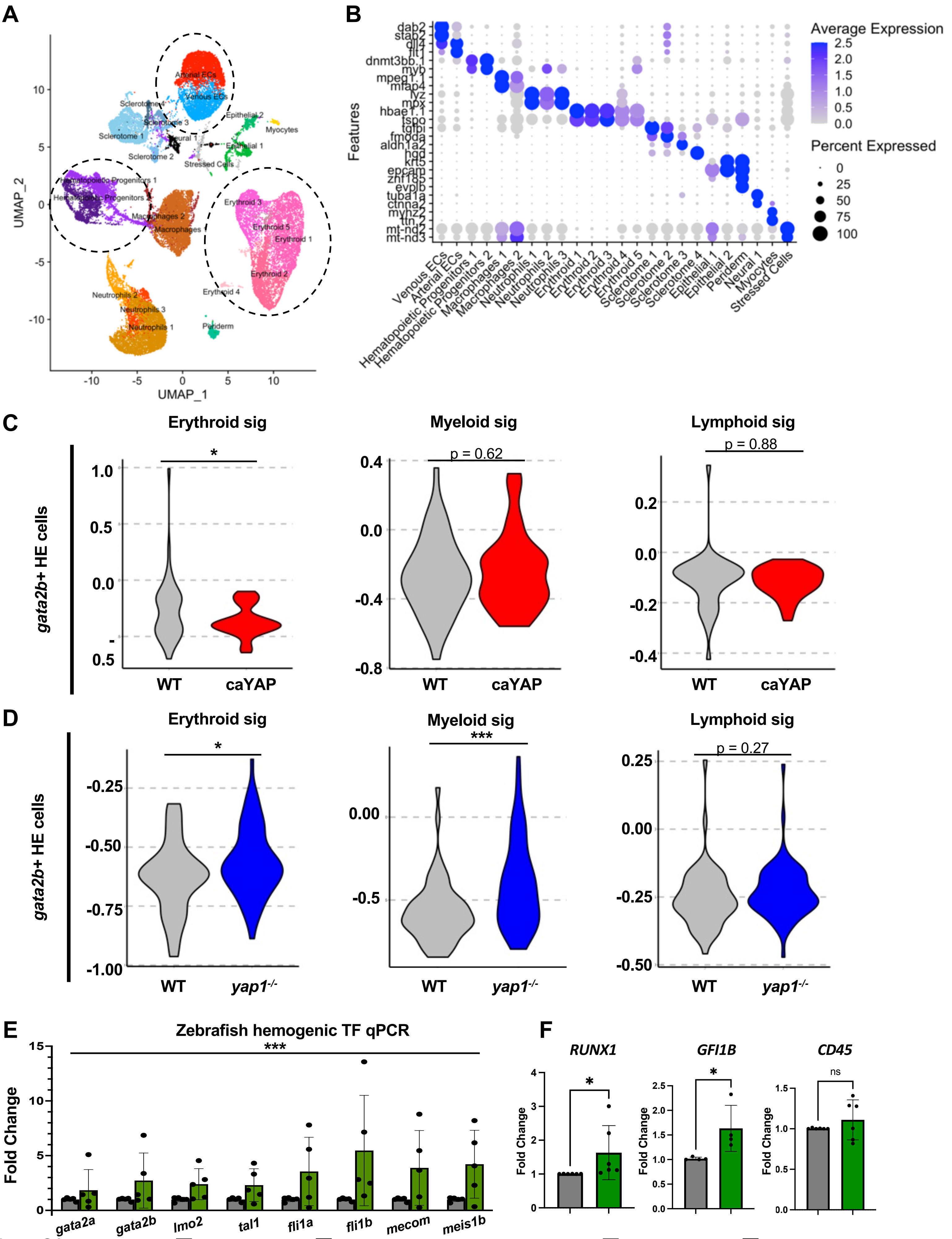
A
UMAP_1
UMAP_2
Arterial ECs
Venous ECs
Sclerotome 1
Sclerotome 2
Sclerotome 3
Sclerotome 4
Epithelial 1
Epithelial 2
Myocytes
Stressed Cells
Hematopoietic Progenitors 1
Hematopoietic Progenitors
Macrophages 2
Macrophages
Erythroid 1
Erythroid 2
Erythroid 3
Erythroid 4
Erythroid 5
Neutrophils 1
Neutrophils 2
Neutrophils 3
Periderm
B
Features
Average Expression
Percent Expressed
dab2
stab2
dll4
flt1
dnmt3bb.1
myb
mpeg1.1
mfap4
lyz
mpx
hbae1.1
tspo
tgfbi
fmoda
aldh1a2
hgd
krt5
epcam
znf185
evplb
tuba1a
ctnna2
mylz2
ttn.2
mt-nd2
mt-nd3
C
Erythroid sig
Myeloid sig
Lymphoid sig
gata2b+ HE cells
p = 0.62
p = 0.88
WT
caYAP
D
Erythroid sig
Myeloid sig
Lymphoid sig
gata2b+ HE cells
p = 0.27
WT
yap1-/-
E
Zebrafish hemogenic TF qPCR
Fold Change
gata2a
gata2b
lmo2
tal1
fli1a
fli1b
mecom
meis1b
DMSO
Yoda1
F
RUNX1
GFI1B
CD45
Fold Change
ns
DMSO
Yoda1

Figure S4

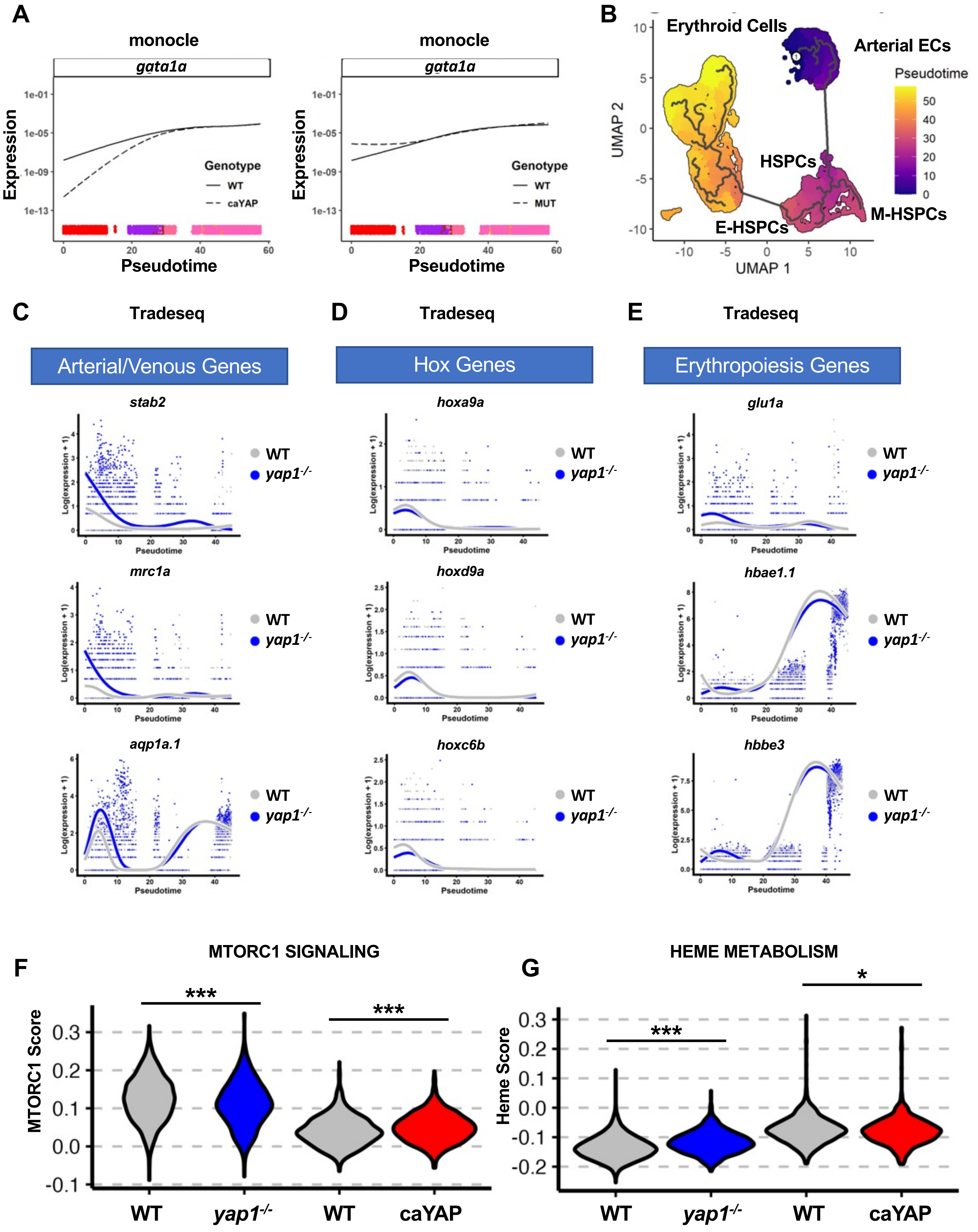
A
monocle
gata1a
Expression
Pseudotime
Genotype
WT
caYAP
MUT
B
Erythroid Cells
Arterial ECs
Pseudotime
HSPCs
E-HSPCs
M-HSPCs
UMAP 1
UMAP 2
C
Tradeseq
Arterial/Venous Genes
stab2
mrc1a
aqp1a.1
D
Hox Genes
hoxa9a
hoxd9a
hoxc6b
E
Erythropoiesis Genes
glu1a
hbae1.1
hbbe3
Log(expression + 1)
WT
yap1-/-
F
MTORC1 SIGNALING
MTORC1 Score
***
WT
yap1-/-
WT
caYAP
G
HEME METABOLISM
Heme Score
*

Figure S5

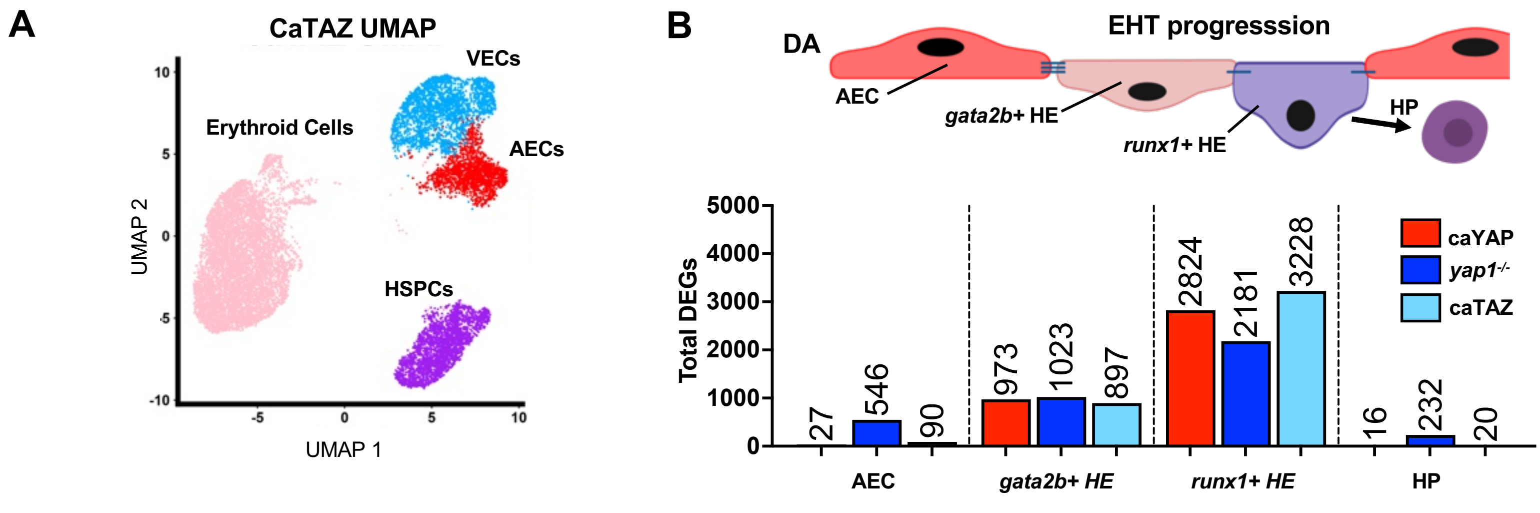
A
CaTAZ UMAP
VECs
Erythroid Cells
AECs
HSPCs
UMAP 2
UMAP 1
10
5
0
-5
-10
B
EHT progresssion
DA
AEC
gata2b+ HE
runx1+ HE
HP
Total DEGs
5000
4000
3000
2000
1000
0
27
546
90
973
1023
897
2824
2181
3228
16
232
20
caYAP
yap1-/-
caTAZ
AEC
gata2b+ HE
runx1+ HE
HP

Figure S6

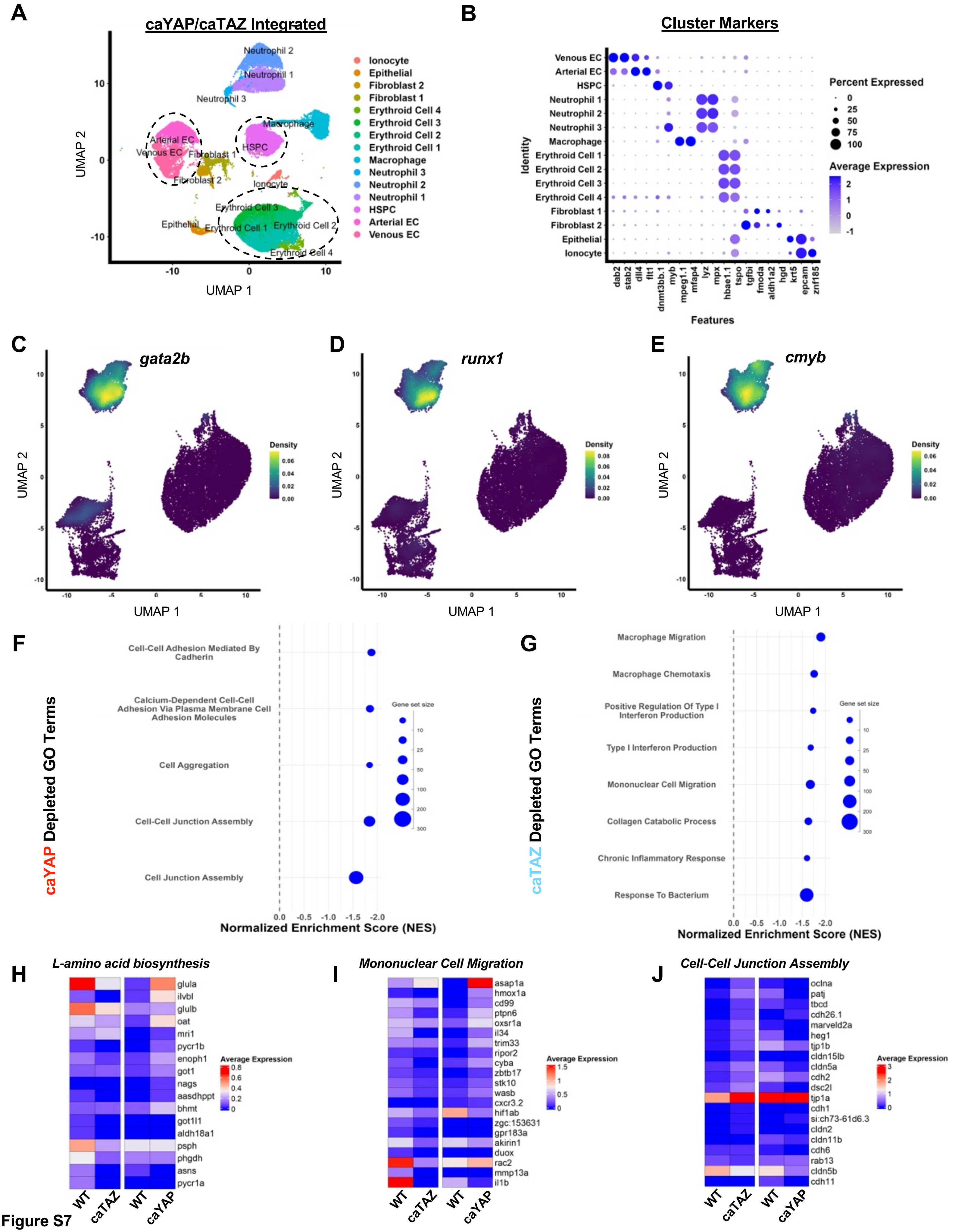
A
caYAP/caTAZ Integrated
UMAP 1
UMAP 2
Neutrophil 2
Neutrophil 1
Neutrophil 3
Macrophage
Arterial EC
Venous EC
HSPC
Fibroblast 1
Fibroblast 2
Ionocyte
Erythroid Cell 3
Epithelial
Erythroid Cell 1
Erythroid Cell 2
Erythroid Cell 4
Ionocyte
Epithelial
Fibroblast 2
Fibroblast 1
Erythroid Cell 4
Erythroid Cell 3
Erythroid Cell 2
Erythroid Cell 1
Macrophage
Neutrophil 3
Neutrophil 2
Neutrophil 1
HSPC
Arterial EC
Venous EC
B
Cluster Markers
Identity
Features
dab2 stab2 dll4 flt1 dnmt3bb.1 myb mpeg1.1 mfap4 lyz mpx hbae1.1 tspo tgfbi fmoda aldh1a2 hgd krt5 epcam znf185
Percent Expressed
0
25
50
75
100
Average Expression
2
1
0
-1
C
gata2b
D
runx1
E
cmyb
UMAP 1
UMAP 2
Density
F
caYAP Depleted GO Terms
Cell-Cell Adhesion Mediated By Cadherin
Calcium-Dependent Cell-Cell Adhesion Via Plasma Membrane Cell Adhesion Molecules
Cell Aggregation
Cell-Cell Junction Assembly
Cell Junction Assembly
Gene set size
Normalized Enrichment Score (NES)
G
caTAZ Depleted GO Terms
Macrophage Migration
Macrophage Chemotaxis
Positive Regulation Of Type I Interferon Production
Type I Interferon Production
Mononuclear Cell Migration
Collagen Catabolic Process
Chronic Inflammatory Response
Response To Bacterium
Gene set size
Normalized Enrichment Score (NES)
H
L-amino acid biosynthesis
glula
ilvbl
glulb
oat
mri1
pycr1b
enoph1
got1
nags
aasdhppt
bhmt
got1l1
aldh18a1
psph
phgdh
asns
pycr1a
Average Expression
WT caTAZ WT caYAP
I
Mononuclear Cell Migration
asap1a
hmox1a
cd99
ptpn6
oxsr1a
il34
trim33
ripor2
cyba
zbtb17
stk10
wasb
cxcr3.2
hif1ab
zgc:153631
gpr183a
akirin1
duox
rac2
mmp13a
il1b
Average Expression
WT caTAZ WT caYAP
J
Cell-Cell Junction Assembly
ocln a
patj
tbcd
cdh26.1
marveld2a
heg1
tjp1b
cldn15lb
cldn5a
cdh2
dsc2l
tjp1a
cdh1
si:ch73-61d6.3
cldn2
cldn11b
cdh6
rab13
cldn5b
cdh11
Average Expression
WT caTAZ WT caYAP

Figure S7

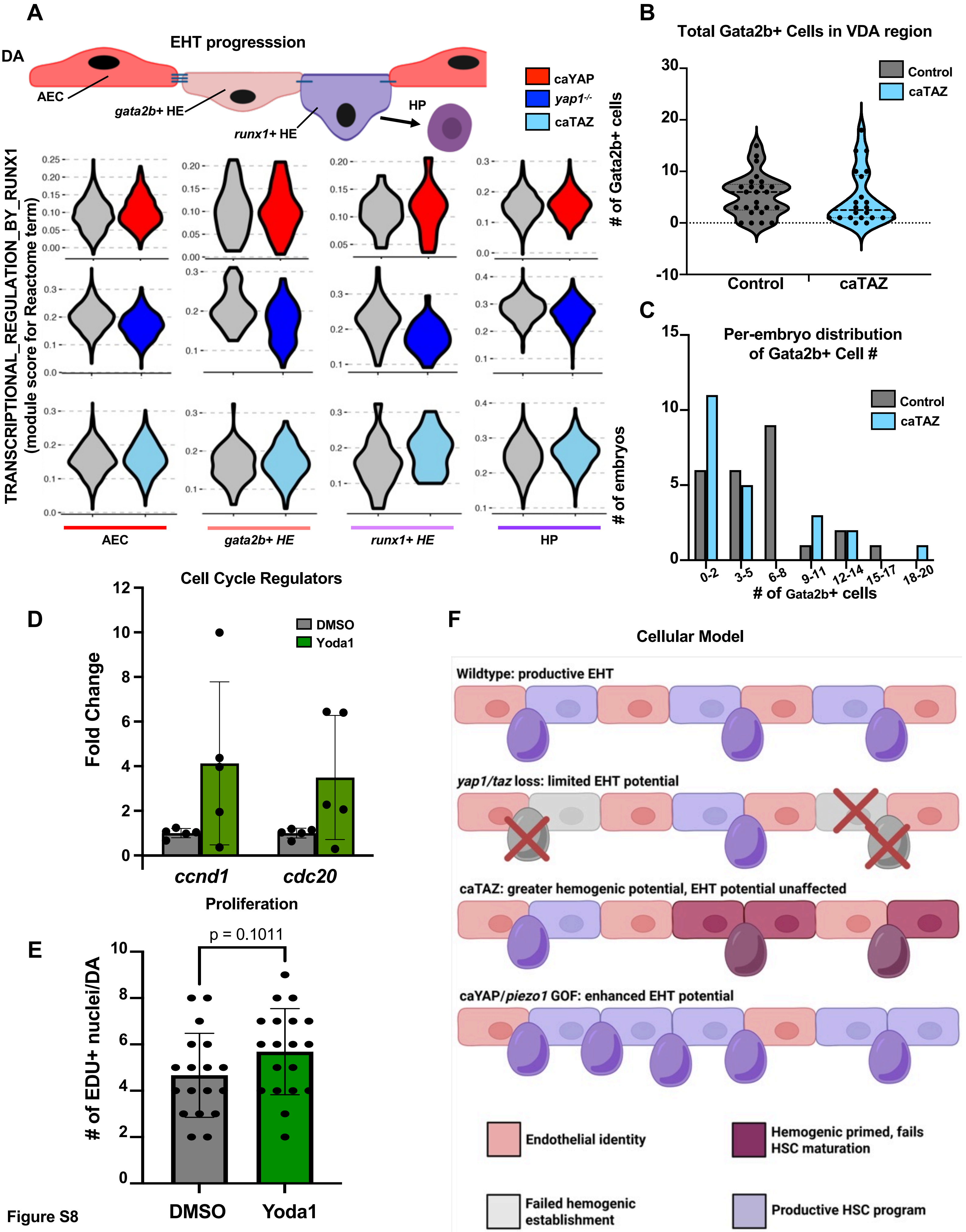
A
EHT progresssion
DA
AEC
gata2b+ HE
runx1+ HE
HP
caYAP
yap1-/-
caTAZ
TRANSCRIPTIONAL_REGULATION_BY_RUNX1
(module score for Reactome term)
AEC
gata2b+ HE
runx1+ HE
HP
B
Total Gata2b+ Cells in VDA region
# of Gata2b+ cells
Control
caTAZ
C
Per-embryo distribution
of Gata2b+ Cell #
# of embryos
# of Gata2b+ cells
0-2
3-5
6-8
9-11
12-14
15-17
18-20
D
Cell Cycle Regulators
Fold Change
DMSO
Yoda1
ccnd1
cdc20
E
Proliferation
p = 0.1011
# of EDU+ nuclei/DA
DMSO
Yoda1
F
Cellular Model
Wildtype: productive EHT
yap1/taz loss: limited EHT potential
caTAZ: greater hemogenic potential, EHT potential unaffected
caYAP/piezo1 GOF: enhanced EHT potential
Endothelial identity
Hemogenic primed, fails HSC maturation
Failed hemogenic establishment
Productive HSC program

Figure S8
